# Premature CD4^+^ T Cell Aging and Its Contribution to Lymphopenia-Induced Proliferation of Memory Cells in Autoimmune-Prone Non-Obese Diabetic Mice

**DOI:** 10.1371/journal.pone.0089379

**Published:** 2014-02-26

**Authors:** Ting-Ting Sheu, Bor-Luen Chiang, Jui-Hung Yen, Wen-Chi Lin

**Affiliations:** 1 Department of Immunology, Tzu Chi University, Hualien, Taiwan, Republic of China; 2 Institute of Microbiology, Immunology and Biochemistry, Tzu Chi University, Hualien, Taiwan, Republic of China; 3 Graduate Institute of Immunology, National Taiwan University, Taipei, Taiwan, Republic of China; 4 Department of Medical Research, National Taiwan University Hospital, Taipei, Taiwan, Republic of China; 5 Department of Molecular Biology and Human Genetics, Tzu Chi University, Hualien, Taiwan, Republic of China; Université Paris Descartes, France

## Abstract

Lymphopenia-induced proliferation (LIP), a mechanism to maintain a constant number of T cells in circulation, occurs in both normal aging and autoimmune disease. The incidence of most autoimmune diseases increases with age, and premature CD4^+^ T cell aging has been reported in several autoimmune diseases. In this study, we tested the hypothesis that premature CD4^+^ T cell aging can cause autoimmune disease by examining whether premature CD4^+^ T cell aging exists and causes LIP in our mouse model. Non-obese diabetic (NOD) mice were used because, in addition to Treg defects, the LIP of T cells has been shown to plays a causative role in the development of insulin-dependent diabetes mellitus (IDDM) in these mice. We found that with advancing age, NOD mice exhibited an accelerated decrease in the number of CD4^+^ T cells due to the loss of naïve cells. This was accompanied by an increase in the percentage of memory cells, leading to a reduced naïve/memory ratio. In addition, both the percentage of CD28^+^ cells in CD4^+^ T cells and IL-2 production decreased, while the percentage of FAS^+^CD44^+^ increased, suggesting that NOD mice exhibit premature CD4^+^ T cell aging. This process preferentially contributed to LIP of memory cells. Therefore, our results suggest that premature CD4^+^ T cell aging underlies the development of IDDM in NOD mice. Given that CD28 and IL-2 play important roles in Treg function, the relationships between premature CD4^+^ T cell aging and lymphopenia as well as Treg defects in autoimmune-prone NOD mice are proposed.

## Introduction

In lymphopenia, when the number of circulating lymphocytes is reduced due to recent infection, leukemia, or treatment with certain cytotoxic medications, an autoproliferation mechanism known as lymphopenia-induced proliferation (LIP) works to maintain the T cell number at a constant level. With aging, reduced thymic output of naïve T cells will also trigger LIP to maintain homeostasis in humans [1]. Lymphopenia is also observed in aged Balb/c mice [2].

In addition to normal physiological situations, LIP can also occur in autoimmune disease states characterized by reduced thymic output or induction of lymphopenia. It has been clearly demonstrated that LIP of T cells occurs and plays a causative role in the development of autoimmune insulin-dependent diabetes mellitus (IDDM) in non-obese diabetic (NOD) mice [3]. LIP also occurs in a murine model of rheumatoid arthritis (RA) [4], and in humans, LIP has been associated with clinical autoimmune diseases [5]. Interestingly, although regulatory T cells (Tregs) are recognized as a major regulator of autoimmune diseases including IDDM in NOD mice [6]–[8], it has been suggested that both lymphopenia and Treg defects are precursors leading to autoimmune diseases [9], [10].

Another characteristic associated with autoimmune diseases is premature aging in CD4^+^ T/T cell compartment (hereafter referred to as premature CD4^+^ T/T cell aging). The incidence of most autoimmune diseases in humans increases with age [11]. Many autoimmune diseases such as multiple sclerosis (MS) and rheumatoid arthritis (RA) occur in the post-menopausal adult and in the elderly, when immune system function is declining [5]. It has been shown that premature T cell aging is not only associated with late-onset autoimmune diseases such as MS [12] and RA [5], [12], [13] but is also associated with early-onset autoimmune diseases such as juvenile idiopathic arthritis [14] and myelodysplastic syndrome [15]. While no causative role of immune aging has been shown in autoimmunity, the fact that autoimmune diseases are caused by dysfunction of the immune system suggests that premature immune aging could lead to autoimmune diseases.

Immunosenescence is an age-related decrease in both innate and adaptive immune functions [16]. In the adaptive immune system, it has been argued that the loss of CD4^+^ helper T (Th) cell function is the pivotal factor in immunosenescence. During aging in both humans and mice, one of the most dramatic changes in the CD4^+^ T cell compartment is a decrease in naïve T cells [17]–[20]. Naïve T cells become memory T cells following antigen stimulation. Therefore, in older individuals, the CD4^+^ T cell subset largely comprises memory cells, while younger individuals have a more balanced representation of both naïve and memory CD4^+^ T cells [20]. Additionally, the T cell signal transduction pathways become increasingly altered with age [21]. Therefore, there are intrinsic defects in the naïve CD4+ T cells from aged mice [22], and CD4^+^ T cell aging markers also include defects in activation, differentiation and expansion after stimulation, altered cytokine production and apoptosis induction of CD4^+^ T cells. Among these defects, loss of expression of the important costimulatory molecule CD28 for activation [17], [19], [23], [24] and reduced production of IL-2, a cytokine for T cell proliferation [18], [22], [23] are the most noticeable age-associated changes. Increased expression of death receptor FAS is also reported in both aged humans and aged mice [25], [26]. Of note, the alterations in CD28 expression and IL-2 production are demonstrated to play important roles in many autoimmune diseases [27]–[30].

Interestingly, CD4^+^ T/T-cell lymphopenia can be observed in the spleens of aged humans [31] and aged Balb/c mice [2], when the function of the immune system is declining. Given that CD4^+^ T/T-cell lymphopenia can induce LIP to cause autoimmune diabetes in NOD mice, it further supports the hypothesis that premature CD4^+^ T cell aging can cause autoimmune disease. No causative role of CD4^+^ T cell aging has been shown in autoimmunity. We first tested this hypothesis by examining whether premature CD4^+^ T cell aging exists in NOD mice and whether this can mediate LIP. Our results demonstrate the presence of premature CD4^+^ T cell aging and its preferential contribution to LIP of memory cells in autoimmune disease-prone NOD mice, and suggest that premature CD4^+^ T cell aging underlies the development of autoimmunity.

## Materials and Methods

### Ethics Statement

Normal 5- to 8-week-old female BALB/c mice were purchased from the National Laboratory Animal Center (Taipei, Taiwan). Female NOD/Lt mice used in this study were bred and maintained in the Laboratory Animal Center at Tzu-Chi University (Hualien, Taiwan). This study was carried out with the recommendations in the guide for the Care and Use of Laboratory Animals of Tzu Chi University. The protocol was approved by the Institutional Animal Care and Use Committee of Tzu Chi University (Permit Number: 94-A-59). Mice used in this study were sacrificed by cervical dislocation, and all efforts were made to minimize suffering. Animals were housed in a specific pathogen-free facility with an individual ventilation cage system at the Laboratory Animal Center. Mice had free access to autoclaved water and food, were maintained at room temperature (∼25°C) on a 12-h light:dark cycle, and were allowed to acclimatize for at least 1 week prior to use. Balb/c mice with tumors or signs of clinical disease were excluded from the study. In some experiments, analysis was performed in 4 different groups according to the following age ranges: Group I, 8–11 weeks old; Group II, 12–15 weeks old; Group III, 16–19 weeks old; and Group IV, 20–23 weeks old.

### Reagents

FITC-conjugated anti-CD3, APC-conjugated anti-CD4, APC-conjugated anti-CD8, and their corresponding isotype controls were purchased from eBioscience (San Diego, CA). PE-Cy5-conjugated anti-CD4, PE-Cy5-conjugated anti-CD8, FITC-conjugated anti-CD44, and their corresponding isotype controls, were obtained from BioLegend (San Diego, CA). PE-conjugated anti-CD45RB, PE-conjugated anti-Fas, and their corresponding isotype controls were purchased from BD Bioscience (San Jose, CA). FITC-conjugated anti-CD28 and its corresponding isotype control were purchased from Serotec (Oxfordshire, UK). Rabbit polyclonal primary anti-Ki-67 and its isotype control were purchased from Abcam (Cambridge, UK) and Santa Cruz Biotechnology (Santa Cruz, CA), respectively. PerCP-conjugated donkey anti-rabbit IgG antibody was obtained from Santa Cruz Biotechnology. T cell mitogen concanavalin A (Con A) was obtained from Sigma (St. Louis, MO).

### Splenocyte isolation

After mice were euthanized by cervical dislocation, spleens (except where otherwise indicated, 2 spleens pooled per experiment) were aseptically removed and placed in sterile RPMI 1640 medium (Sigma) supplemented with 10% FBS, penicillin (1×10^5^ units/L), and streptomycin (100 mg/L) (Sigma). This medium is referred to as complete RPMI. Single-cell suspensions were prepared by gently disrupting spleens with the plunger end of a syringe. Splenocytes were isolated via centrifugation (400×*g*), and red blood cells were lysed using ammonium chloride reagent at room temperature for 5 min, following which an equal volume of 1× PBS was added and cells were centrifuged again at 400×*g*. Splenocytes were then washed with 1× PBS and suspended in complete RPMI. Cell numbers were determined by exclusion of 0.4% trypan blue using a hemocytometer.

### IL-2 assay

Splenocytes (5×10^5^ cells/well) isolated from Balb/c and NOD mice were stimulated with Con A (2.5 µg/mL) in 96-well flat-bottom plates. After 20 hours, culture supernatants were collected and IL-2 levels were determined by enzyme-linked immunosorbent assay (ELISA) (R & D System, MN).

### Flow cytometry

The surface expression of CD3, CD4, CD8, CD44, CD45RB, CD28, and FAS, as well as the intracellular expression of Ki-67 in splenocytes was determined by staining cells with specific antibodies conjugated to different fluorophores and analyzing them with a FACSCalibur flow cytometer (BD Biosciences, San Jose, CA). All staining steps were performed at 4°C in the dark for 20 min. FCS Express V3 software (De Novo Software Inc, Thornhill, Canada) was used to perform 1-, 2-, 3-, or 4-color analysis of the data. Except where otherwise indicated, 10,000 cells were collected and analyzed for each sample.

### CD3 and CD4 expression

Splenocytes were stained with APC-conjugated anti-CD4 and FITC-conjugated anti-CD3 in FACS buffer (1× PBS with 1% BSA and 0.1% NaN_3_). APC-conjugated and FITC-conjugated mice IgG were used as isotype-matched background controls.

### CD44 and CD45RB expression

Splenocytes were stained with FITC-conjugated anti-CD44, PE-conjugated anti-CD45RB, and PE-Cy5-conjugated anti-CD4 or anti-CD8 in FACS buffer. FITC-conjugated, PE-conjugated, and PE-Cy5-conjugated mouse IgGs were used as isotype-matched background controls.

### CD28 expression after stimulation

Splenocytes (5×10^5^ cells/well) isolated from Balb/c and NOD mice were stimulated with Con A (2.5 µg/mL) in 96-well flat-bottom plates. After 20 hours, cells were harvested and stained with APC-conjugated anti-CD4 and FITC-conjugated anti-CD28 in FACS buffer. APC-conjugated and FITC-conjugated mouse IgGs were used as isotype-matched background controls.

### Fas expression

Splenocytes were stained with FITC-conjugated anti-CD44, PE-conjugated anti-Fas, and PE-Cy5-conjugated anti-CD4 or anti-CD8 in FACS buffer. FITC-conjugated, PE-conjugated, and PE-Cy5-conjugated mouse IgGs were used as isotype-matched background controls.

### Ki-67 expression in naïve and memory cells

Splenocytes were simultaneously stained with APC-conjugated anti-CD4, FITC-conjugated anti-CD44, and PE-conjugated anti-CD45RB or isotype control antibodies in 96-well flat-bottom plates. To determine intracellular Ki-67 expression, cells were fixed in 2% paraformaldehyde/0.3% saponin/PBS and were then stained with rabbit anti-Ki-67 primary antibody followed by Per-CP-conjugated anti-rabbit secondary antibody in 0.3% saponin/FACS buffer.

### Statistical analysis

The Mann-Whitney test was used to make comparisons between the 2 independent samples, that is, normal Balb/c mice and NOD mice. The effect of age or naïve/memory ratio of CD4^+^ T cells on cell number or percentage of T cell subsets was analyzed using the regression option in Minitab Release 13 for Windows (www.minitab.com; State College, PA). P<0.05 was considered significant.

## Results

### The numbers of T cells and CD4^+^ T cells decreased with age in normal and NOD mice

**Figure 1:**
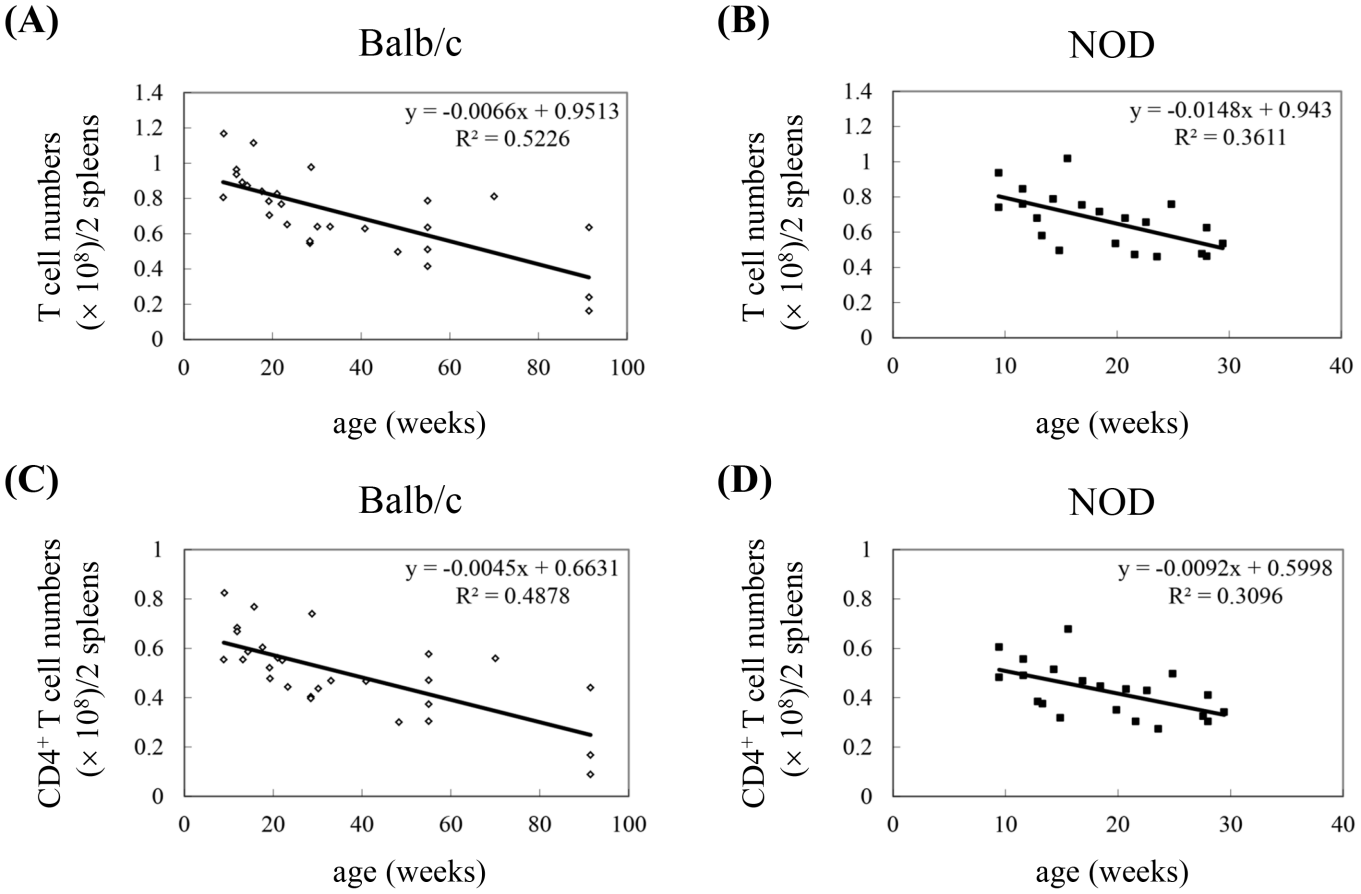
Dynamic lymphopenia of T cells and CD4^+^ T cells in the spleens of NOD mice compared to control Balb/c mice. (A) CD3^+^ T cells in spleens of Balb/c mice. (B) CD3^+^ T cells in spleens of NOD mice. (C) CD4^+^ T cells in spleens of Balb/c mice. (D) CD4^+^ T cells in spleens of NOD mice. The results of each experiment were obtained from 2 pooled spleens (Balb/c: n = 54 mice, 4 spleens at 91 weeks old; NOD: n = 40 mice, 2 spleens at 28 weeks old). Gating was performed on live cells. The total numbers of T cells (CD3^+^) and CD4^+^ T cells were calculated by multiplying their percentages in the spleen by the total cell number of splenocytes. The regression equation is shown in each panel.

A reduction in the number of all T cells and CD4^+^ T cells specifically is an important characteristic of immune aging. To determine whether T cell aging occurs in an animal model of type 1 DM, we compared the number of total T cells and CD4^+^ T cells in the spleens of NOD mice to the number in the spleens of normal Balb/c mice. Both Balb/c (8–91 weeks of age) and NOD mice (8–30 weeks of age) exhibited an inverse correlation between age and the number of splenic T cells and CD4^+^ T cells, and the decrease in T cell number was accelerated in NOD mice, as indicated by the Pearson correlation coefficient (r_p_) (Table 1) and the slope (Figure 1). At 9 weeks of age, the number of splenic T cells in NOD mice was slightly less than in control mice, and this difference increased with increasing age of the mice (Figure 1 and Table 1). The reduction in T cells in NOD mice compared to Balb/c mice was mainly due to the loss of CD4^+^ T cells (Figure 1D). The number of splenic CD4^+^ T cells in NOD mice at 20 weeks of age was similar to that of Balb/c mice at 53 weeks of age. Because reduced CD4^+^ T cell number is a characteristic of immune aging, these results suggest that NOD mice undergo a process of premature immune aging or at least premature T cell aging.

**Table 1:** Correlation between T cell number, naïve/memory cell ratio, and age in Balb/c and NOD mice.

	Balb/c mice,				NOD mice	
	chronological age				chronological age	
	W8∼W91		W8∼W30		W8∼W30	
	r_p_ b	*P*	r_p_	*P*	r_p_	*P*
T cell subset^a^						
CD3+	−0.723	<0.001	-	-	−0.551	0.006
CD3+CD4+	−0.698	<0.001	-	-	−0.495	0.016
CD4+	−0.642	<0.001	−0.304	0.109	−0.465	0.005
CD4+CD45RB+CD44-	−0.798	<0.001	−0.345	0.067	−0.363	0.032
CD4+CD45RB-CD44+	0.399	0.008	0.274	0.150	−0.056	0.750
Naïve/Memory ratio	−0.818	<0.001	−0.571	0.001	−0.318	0.062

a Number of cells (×10^8^)/ml in 2 spleens.

b Pearson correlation coefficient.

### Naïve but not memory T cell number decreases in NOD mice compared to Balb/c mice

**Figure 2:**
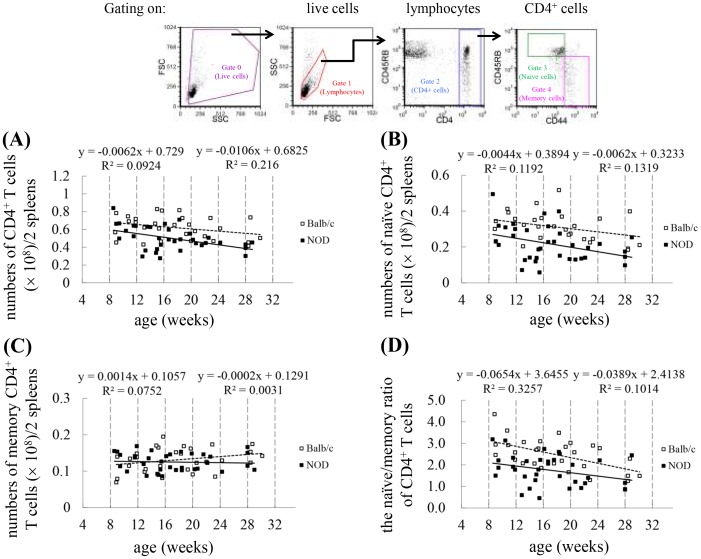
Reduction in naïve cells but not in memory cells in splenic CD4^+^ T cells of NOD mice compared to Balb/c mice. Splenocytes isolated from Balb/c and NOD mice were stained with PE-Cy5-conjugated anti-CD4, FITC-conjugated anti-CD44, and PE-conjugated anti-CD45RB. Gating was performed on live CD4^+^ T cells. Naïve cells were defined as the CD45RB^+^CD44^−^ phenotype, whereas memory cells were defined with CD45RB^−^CD44^+^. Dot plots are shown for the number of CD4^+^ T cells (A), the number of naïve CD4^+^ T cells (B), the number of memory CD4^+^ T cells (C), and the ratio of naïve/memory cells in CD4^+^ T cells (D) in the spleen of Balb/c and NOD mice. The results of each experiment were obtained from 2 pooled spleens (Balb/c: n = 58 mice; NOD: n = 68 mice, 4 spleens at 28 weeks old). Shown on the top is the gating strategy used in the analysis of the cell numbers of various T cell subsets. The number of cells in each T cell subset was calculated by multiplying their percentages in the spleen by the total cell number of splenocytes. The regression equation is shown in each panel (left: Balb/c; right: NOD).

In older animals, the CD4^+^ subset largely comprises memory T cells, while younger individuals have a more balanced representation of both memory and naïve CD4^+^ T cells [32]. To further explore the phenomenon of premature immune/T cell aging in NOD mice, we examined the number of total, naïve, and memory CD4^+^ T cells as well as the naïve/memory ratio in CD4^+^ T cells in mice at various ages. Because 97% of the CD4^+^ lymphocytes in the spleens were T cells, we gated on these cells and considered them to be a T cell population for the remainder of the experiments in the study. As shown in Table 1, in Balb/c mice (8−91 weeks old), age was inversely correlated with the number of total (P<0.001) and naïve CD4^+^ T cells (P<0.001) as well as the naïve/memory CD4^+^ T cell ratio (P<0.001), but was positively correlated with the number of memory CD4^+^ T cells (P = 0.008). When compared to age-matched young control mice (8−30 weeks old), NOD mice exhibited an inverse correlation between CD4^+^ T cell number and age (P = 0.005), while Balb/c mice did not (P = 0.109) (Table 1 and Figure 2A), suggesting the existence of premature T cell aging in these young NOD mice. NOD mice also exhibited an inverse correlation between the number of naive CD4^+^ T cells and age (P = 0.032), but not Balb/c mice (P = 0.067) (Table 1 and Figure 2B). Interestingly, however, the numbers of naïve CD4^+^ T cells in the spleens of NOD mice were much lower when they were 12−15 weeks old than when they were 16−19 weeks old. In addition, the number of memory cells (P = 0.150) (Table 1 and Figure 2C) and the naïve/memory ratio (P = 0.062) (Table 1 and Figure 2C) in NOD mice were not correlated with age. In contrast, in young Balb/c mice (8−30 weeks old), the observed age-related decrease in the naïve/memory CD4^+^ T cell ratio appears to be a better marker for T cell aging (P = 0.001). Therefore, these results suggest that premature T cell aging occurs in NOD mice in a dynamic manner.

The number of total CD4^+^ T cells (P = 0.001) and naïve CD4^+^ T cells (P = 0.0001), as well as the naïve/memory ratio (P = 0.0003), was lower in NOD mice than in control Balb/c mice at 8−30 weeks, whereas the number of memory cells did not differ between the 2 groups (P = 0.3881) (Figure 2 and Table 2). In addition, the percentage of naïve CD4^+^ T cells was significantly lower in NOD mice than in control mice, while the percentage of memory cells was higher. These results suggest that the dynamic premature aging of T cells in NOD mice is largely due to the change in the number of naïve CD4^+^ T cells; therefore, the percentage of naïve T cells or the naïve/memory ratio could be used as a marker for T cell aging in NOD mice. It appears that the process of T cell aging could be occurring as early as 8−9 weeks of age (Figure 2), which is the earliest age tested in this study, and occurs 5−7 weeks before the onset of diabetes started at age of 13−16 weeks old in female NOD mice [3], . In our female NOD colony, the diabetes onset occurs at age of 16 weeks old; the onset rate is 25% between 18 weeks and 20 weeks of age. Therefore, our results suggest that T cell aging occurs before the onset of diabetes.

**Table 2:** Total cell number in T cell subsets, naïve/memory cell ratio, and percentage of naïve and memory cells in Balb/c and NOD mice at 8−30 weeks of age.

	Balb/c		NOD		*P* b
	Mean ± SEM	Median (range)	Mean ± SEM	Median (range)	
T cell subset^a^					
CD4+	0.62±0.02	0.63 (0.42−0.83)	0.52±0.02	0.49 (0.28−0.84)	0.0010
CD4+CD45RB+CD44-	0.31±0.01	0.31 (0.18−0.52)	0.22±0.02	0.21 (0.06−0.49)	0.0001
CD4+CD45RB-CD44+	0.13±0.01	0.13 (0.07−0.19)	0.13±0.00	0.12 (0.09−0.17)	0.3881
Naïve/Memory ratio	2.48±0.13	2.58 (1.24−4.35)	1.75±0.12	1.79 (0.46−3.19)	0.0003
Naïve % in CD4+ T cells	0.50±0.01	0.51 (0.31−0.63)	0.42±0.02	0.42 (0.21−0.59)	0.0015
Memory % in CD4+ T cells	0.21±0.01	0.20 (0.12−0.21)	0.26±0.01	0.24 (0.17−0.46)	0.0030

a Number of cells (×10^8^)/ml in 2 spleens.

b By Mann-Whitney test.

The accumulative onset rate of diabetes reaches 33.3%−70% at age of 23 weeks old [35], [36]. To further test our hypothesis whether premature CD4^+^ T cell aging can cause IDDM in NOD mice, in some experiments, only mice at age of 8−23 weeks old were examined to analyze other T cell aging markers. These mice were divided into 4 different age groups based on the number of naïve CD4^+^ T cells (Figure 2D) since the percentage of naïve T cells could be used as a marker for T cell aging in NOD mice as the following age ranges: Group I, 8−11 weeks old; Group II, 12−15 weeks old; Group III, 16−19 weeks old; and Group IV, 20−23 weeks old.

### CD28 expression is decreased in NOD mice

Given that naïve and memory T cells are different cell populations with unique cellular characteristics, age-associated alterations in T cell function including activation and cytokine production could be secondary to the change in the percentage of naïve and memory T cells [37]. Therefore, it was examined whether there is a correlation between other CD4^+^ T cell aging markers and the percentage of naïve and memory cells in CD4^+^ T cells in each age group.

**Figure 3:**
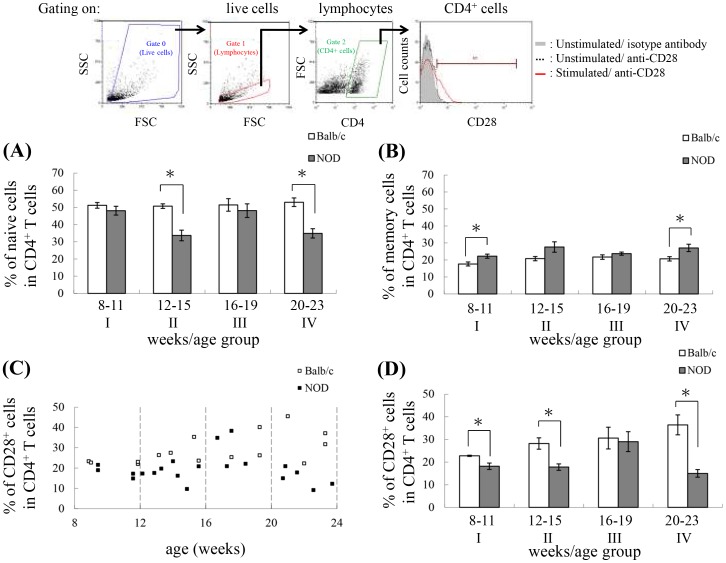
Correlation between age and CD28 expression on CD4^+^ T cells. (A, B) The bar plots show the percentage of naïve CD4^+^ T cells (A) and memory CD4^+^ T cells (B) in each age group (age group I: Balb/c: n = 14 mice; NOD: n = 16 mice; age group II: Balb/c: n = 14 mice; NOD: n = 18 mice; age group III: Balb/c: n = 10 mice; NOD: n = 16 mice; age group IV: Balb/c: n = 10 mice; NOD: n = 10 mice). Data are shown as mean ± SEM. (C, D) Splenocytes isolated from Balb/c and NOD mice were stimulated with Con A, harvested, and stained with APC-conjugated anti-CD4 and FITC-conjugated anti-CD28 antibodies, and then 30,000 cells were analyzed by flow cytometry with gating performed on live CD4^+^ T cells. Shown on the top is the gating strategy used in the analysis of the percentage of CD28^+^ cells in the CD4^+^ T cell population. (C) The dot plot represents the percentage of CD28^+^ cells in the CD4^+^ T cell population with age. Each data point is taken from pooled data for 2 spleens (Balb/c: n = 30 mice; NOD: n = 40 mice). (D) The bar plot shows the percentage of CD28^+^ cells in the CD4^+^ T cell population broken down by age group (age group I: Balb/c: n = 8 mice; NOD: n = 8 mice; age group II: Balb/c: n = 8 mice; NOD: n = 14 mice; age group III: Balb/c: n = 6 mice; NOD: n = 8 mice; age group IV: Balb/c: n = 8 mice; NOD: n = 10 mice). Data are shown as mean ± SEM. *The values for NOD mice were significantly different from the values for Balb/c mice at *P*<0.05.

The percentage of naïve CD4^+^ T cells was significantly lower in NOD mice than in control mice in age groups II (12−15 weeks old; P = 0.0021) and IV (20−23 weeks old; P = 0.02) but not in age groups I and III (Figure 3A). In contrast, the percentage of memory CD4^+^ T cells was significantly higher in NOD mice than in control mice in age groups I (8−11 weeks old; P = 0.0323) and IV (20−23 weeks old; P = 0.0373) (Figure 3B).

Replicative stress in T cells has been associated with progressive loss of expression of the important costimulatory molecule CD28, and this is one of the most noticeable age-associated changes in the human T cell population [38]–[43], thus providing a marker of T cell aging. At birth, CD28 is expressed on more than 99% of peripheral blood T cells [44], whereas the percentage of CD28^−^ T cells can reach as high as 45% in some centenarians [45], [46]. In mice, although the surface expression of CD28 is low without stimulation, mitogen stimulation can increase the percentage of CD28^+^ T cells, and the percentage is higher in T cells from younger mice than in T cells from older mice [47]. To further explore the T cell aging phenomenon in NOD mice, we stimulated splenic CD4^+^ T cells with mitogen Con A and examined surface expression of CD28. As seen in Figure 3C, in general, the percentage of CD28^+^ cells in CD4^+^ T cells isolated from NOD mice is less than that isolated from control Balb/c mice. The difference between control and NOD mice is significant in age groups I, II and IV but not in age group III (Figure 3D). This is consistent with the result shown in Figure 3A and further suggests that NOD mice undergo a dynamic T cell aging process that is correlated with the percentage of naïve CD4^+^ T cells.

### IL-2 production is reduced in NOD mice

**Figure 4:**
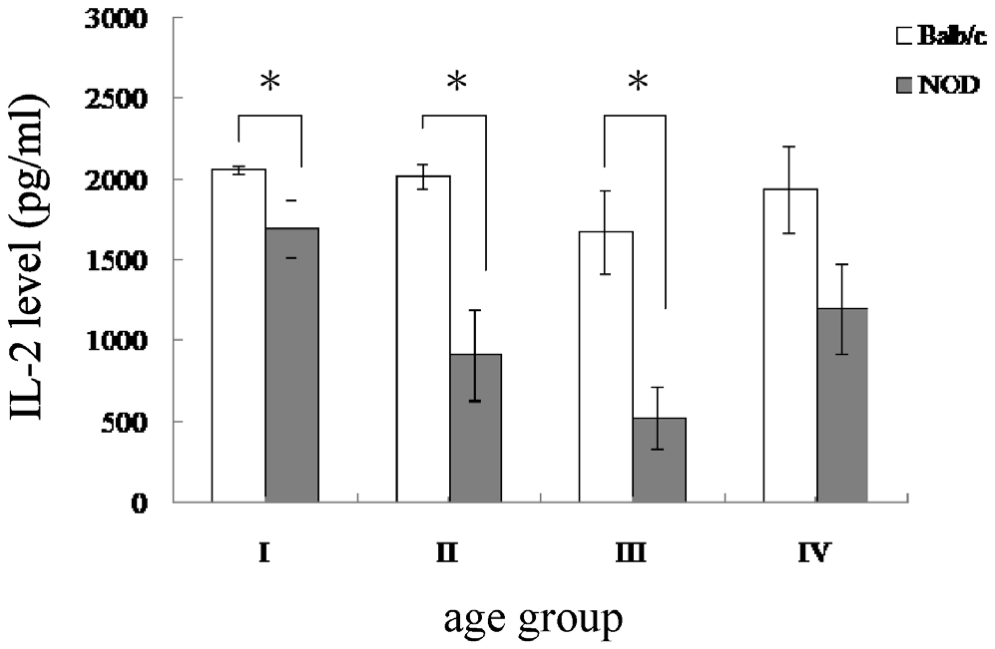
Reduced IL-2 production from splenocytes of NOD mice compared to age-matched Balb/c mice. The bar plots show the IL-2 level in each age group. Data are shown as mean ± SEM (Balb/c: n = 8−10 mice per group; NOD: n = 8−10 mice per group). Splenocytes isolated from Balb/c and NOD mice were stimulated with Con A and culture supernatants were harvested to determine IL-2 levels. *The values for NOD mice were significantly different from the values for Balb/c mice at *P*<0.05.

IL-2 is an important cytokine for T cell proliferation and is mainly produced by activated CD4^+^ T cells. Reduced production of IL-2 from CD4^+^ T cells is evident in older humans and mice, thus providing a marker of T cell aging. IL-2 can mediate biological responses through binding to its high-affinity receptor and this IL-2/IL-2R interaction also leads to endocytosis of this complex to limit IL-2 signal transduction [48]. Therefore, the IL-2 level in culture medium is the net result of both secreting and internalization pathways. To examine IL-2 production with culture medium, a time point when highest IL-2 level reached should be chosen. To further explore the CD4^+^ T cell aging phenomenon in NOD mice, we stimulated splenocytes with Con A for 20 hours and examined IL-2 levels in culture supernatants. We chose 20 hour post-stimulation since it has been shown that IL-2 level in culture medium reaches and maintains on a maximum level between 18 hours and 48 hours post Con A stimulation of splenocytes isolated from young C57BL/6 mice [49]. As shown in Figure 4, in general, the IL-2 production in splenocytes isolated from NOD mice is less than that isolated from control Balb/c mice. The difference between control and NOD mice is significant in age groups I, II and III but not in age groups IV (Figure 4). Interestingly, in age groups I and III the percentage of naïve CD4^+^ T cells is the same in both control and NOD mice, while the reduced IL-2 production in NOD mice suggests that the presence of intrinsic defects may exist in the naïve cells of NOD mice. It has been shown that recent thymic emigrants generated from aged mice possess intrinsic defects and produce less IL-2 [22]. This result suggests that, by comparison with control Balb/c mice, NOD mice undergo a dynamic premature T cell aging process with the presence of intrinsic defects in the naïve cells.

### Cell surface expression of FAS on CD4^+^ T cells

**Figure 5:**
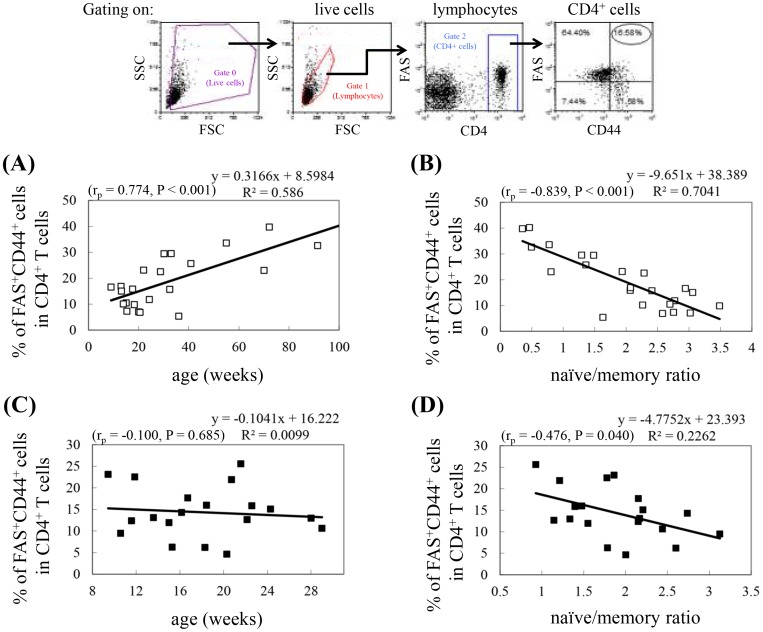
Correlation between the percentage of CD4^+^ T cells that are FAS^+^CD44^+^ and age or the naïve/memory cell ratio. Splenocytes isolated from Balb/c and NOD mice were stained with PE-Cy5-conjugated anti-CD4, FITC-conjugated anti-CD44, and PE-conjugated anti-FAS antibodies. Gating was performed on live CD4^+^ T cells. (A, C) The dot plots represent the percentage of CD4^+^ T cells that are FAS^+^CD44^+^ in Balb/c mice (A) and NOD mice (C) versus age. (B, D) The dot plots represent the percentage of CD4^+^ T cells that are FAS^+^CD44^+^ in Balb/c mice (B) and NOD mice (D) versus naïve/memory ratio, as determined in Figure 2D. The experimental data were obtained from 2 pooled spleens for each experiment (Balb/c: n = 46 mice; NOD: n = 38 mice). Shown on the top is the gating strategy used in the analysis of the percentage of FAS^+^CD44^+^ cells in the CD4^+^ T cell population. Pearson correlation coefficients (r_p_), P values, and regression equations are shown in each panel.

In addition to molecules like CD28, which are important for T cell activation, molecules involved in T cell death also undergo age-related changes. The expression of FAS, a protein that triggers apoptosis when bound to its ligand, increases on human CD4^+^ T cells until approximately 61−74 years of age, and then decreases again [25]. The expression of CD45RO in humans or CD44 in mice is a marker for pre-activated/memory cells that also increases with age. The percentage of FAS^+^CD45RO^+^ T cells in CD4^+^ T cells exhibits the same trend as that observed in the total lymphocyte population [25]. In mice, the percentage of FAS^+^CD44^+^ cells in enriched splenic T cells is also higher in old (26 months) mice than in young (2 months) mice [26]. The percentage of FAS^+^CD44^+^ cells in splenic CD4^+^ T cells from Balb/c mice increased with age up to 91 weeks (Figure 5A) and was inversely correlated with the naïve/memory ratio (Figure 5B). In contrast, the percentage of FAS^+^CD44^+^ in splenic CD4^+^ T cells isolated from NOD mice (8−30 weeks of age) did not increase with advanced age (Figure 5C), probably because NOD mice undergo dynamic but inconsistent T cell aging. Therefore, we analyzed whether there is an inverse correlation between the percentage of FAS^+^CD44^+^ cells in splenic CD4^+^ T cells and the naïve/memory ratio of CD4^+^ T cells. Results of this analysis demonstrated that the percentage of FAS^+^CD44^+^ cells in splenic CD4^+^ T cells was inversely correlated with the naïve/memory ratio of CD4^+^ T cells in NOD mice (P = 0.04; Figure 5D). This result also indicated a dynamic T cell aging process in NOD mice.

### Proliferation rates of naïve and memory CD4^+^ T cells

**Figure 6:**
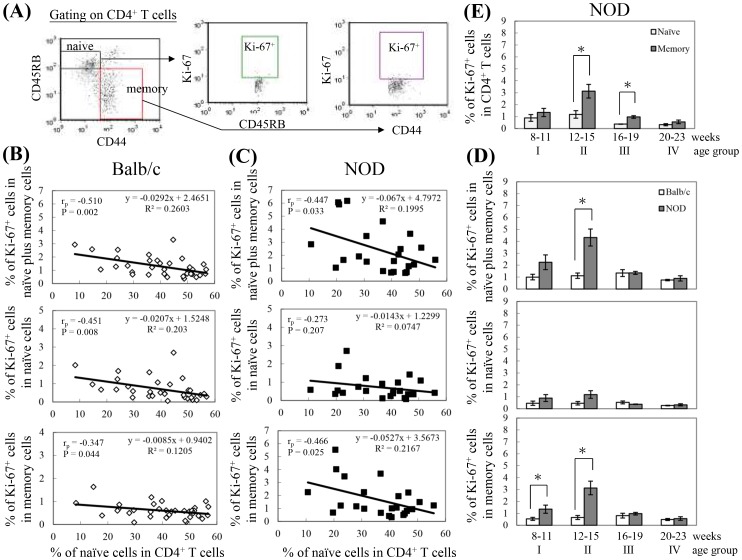
Correlation between the percentage of Ki-67-positive cells in naïve, memory, or naïve plus memory CD4^+^ T cells and the percentage of naïve cells in CD4^+^ T cells. Splenocytes isolated from Balb/c and NOD mice were stained with APC-conjugated anti-CD4, FITC-conjugated anti-CD44, PE-conjugated anti-CD45RB, and Per-CP-conjugated anti-Ki-67, and then 30,000 cells were analyzed with flow cytometry with gating performed on live CD4^+^ T cells, naïve (CD45RB^+^CD44^−^) CD4^+^ T cells, and memory (CD45RB^−^CD44^+^) CD4^+^ T cells (A). (B, C) The dot plots represent correlation between the percentage of Ki-67-positive cells in naïve, memory, and naïve plus memory CD4^+^ T cells and the percentage of naïve cells in CD4^+^ T cells of Balb/c (B) and NOD (C) mice. Data for each experiment were pooled for 2 spleens (Balb/c: n = 66 mice; NOD: n = 46 mice). Pearson correlation coefficients (r_p_), P values, and the regression equation are shown in each panel. (D) The bar plots show the percentage of Ki-67-positive cells in naïve, memory, and naïve plus memory CD4^+^ T cells in each age group. Each point represents the mean ± SEM for data obtained from 3 to 7 experiments (age group I: Balb/c: n = 12 mice; NOD: n = 8 mice; age group II: Balb/c: n = 10 mice; NOD: n = 14 mice; age group III: Balb/c: n = 12 mice; NOD: n = 10 mice; age group IV: Balb/c: n = 6 mice; NOD: n = 8 mice). *The values for NOD mice were significantly different from the values for the Balb/c mice at *P*<0.05. (E) The bar plots show the percentage of Ki-67-positive cells in naïve and memory CD4^+^ T cells in each age group of NOD mice. Each point represents the mean ± SEM. *The values for memory cells were significantly different from the values for naïve cells at *P*<0.05.

LIP of CD4^+^ and CD8^+^ T cells in NOD mice generates IDDM [3]. Since LIP can occur in both naïve and memory cells, we investigated whether LIP is correlated with immune aging, as indicated by a reduction in the percentage of naïve CD4^+^ T cells. Ki-67 was used to detect proliferating cells because it is expressed intracellularly during the G_1_ and M phases and disappears in the G_0_ phase of the cell cycle [50]. In populations of naïve plus memory cells in Balb/c mice, the percentage of proliferating cells is inversely correlated with the percentage of naïve cells (P = 0.002; Figure 6B, top panel). This inverse correlation occurs in both the population with the naïve phenotype (P = 0.008; Figure 6B, middle panel) and the population with the memory phenotype (P = 0.044; Figure 6B, bottom panel). In Balb/c mice, naïve cells respond better to the reduction in naïve cells than do memory cells, given that the slope of the regression equation is −0.0207 for naïve cells (Figure 6B, middle panel) and −0.0085 for memory cells (Figure 6B, bottom panel). It has been shown that under acutely lymphopenic conditions (e.g., sublethal irradiation), LIP of most naïve cells occurs at a slow rate and the cells retain their naïve phenotype [51], [52]. This slow expansion of naïve cells is termed homeostatic proliferation (HP) and is mediated by low-affinity self-peptides/MHCs and an increased concentration of IL-7/IL-15 resulting from the reduction in T cell number [53]–[56].

In NOD mice, the percentage of proliferating cells in populations containing both naïve and memory cells was also inversely correlated with the percentage of naïve cells (P = 0.033; Figure 6C, top panel). However, when comparing the same percentage of naïve cells, the proliferation rate was higher in NOD mice than in Balb/c mice (Figure 6B and 6C, top panels). This difference in LIP between Balb/c and NOD mice, at a given percentage of naïve cells, is due to the higher percentage of proliferating memory cells (Figure 6B and 6C, bottom panels), but not naïve cells, in NOD mice (Figure 6B and 6C, middle panels). Therefore, our results clearly demonstrate that, in both Balb/c and NOD mice, LIP is correlated with T cell aging. In addition, in NOD mice, the rate of LIP in cells with memory phenotype was higher than in Balb/c mice.

Since the onset of IDDM in NOD mice occurs at a young age, that is, from 13 to 16 weeks of age [3], , we next analyzed LIP of CD4^+^ T cells in Balb/c and NOD mice in various age groups ranging from 8 to 23 weeks. There was a trend toward higher LIP of CD4^+^ T cells in NOD than in Balb/c mice in age groups I and II, with significance only in age group II, but no difference in age group III and IV (Figure 6D, top panel). This result correlates with the percentage of naïve CD4^+^ T cells in age groups I, II, and III. In age group IV, although there was a significantly lower percentage of naïve cells in NOD mice than in Balb/c mice, LIP was low, implying that the cells might enter a phase of replicative senescence after vigorous proliferation at younger ages, or that lymphopenia alone is not enough to induce LIP in age group IV NOD mice. This difference in LIP was mainly due to the LIP difference in cells with memory phenotype rather than in cells with naïve phenotype (Figure 6D, bottom and middle panels). In addition, in NOD mice, the level of LIP in cells with memory phenotype was higher than that in cells with naïve phenotype, with significance in age group II (P = 0.0152) and III (P = 0.0122) (Figure 6E). Taken together, these results indicate that premature T cell aging that occurred in young NOD mice could preferentially contribute to lymphopenia-induced proliferation in cells with memory phenotype and thus contribute to the pathogenesis of IDDM.

## Discussion

Although the phenomenon of immune aging has been seen in several autoimmune settings, our study showed that premature aging of T cells in the murine NOD model can directly cause LIP of cells with the memory phenotype (Fig 6C), thus mediating the onset of IDDM. This study examined several parameters as indicators of premature T cell aging in NOD mice compared to age-matched control Balb/c mice, including the number of total, naïve, and memory CD4^+^ T cells in the spleen; the percentage of naïve and memory cells in CD4^+^ T cells; the ratio of naïve to memory CD4^+^ T cells; IL-2 production and the percentage of CD28^+^ cells in CD4^+^ T cells after Con A stimulation; the percentage of FAS^+^CD44^+^ cells in CD4^+^ T cells; and lymphopenia-induced proliferation [1]. We found that with advanced age, NOD mice showed an accelerated decrease in the number of CD4^+^ T cells, primarily due to the loss of naïve cells, and this propensity was more significant in NOD mice from age groups II and IV. In contrast, the percentage of memory cells in the CD4^+^ T cell population increased slightly, leading to a reduced naïve/memory cell ratio. The decrease in IL-2 production and in the percentage of CD28^+^ cells in CD4^+^ T cells after Con A stimulation, as well as the alteration in the percentage of FAS^+^CD44^+^ cells in CD4^+^ T cells, also suggested that NOD mice exhibit premature CD4^+^ T cell aging. In addition, the LIP level in control Balb/c mice was inversely correlated with the percentage of naïve cells. Compared to age-matched control mice, the LIP level of NOD mice in age group II was significantly higher, also suggesting premature CD4^+^ T cell aging phenomenon.

Stimulation with Con A was performed in this study was due to that, not only reduced CD28 expression after Con A stimulation in aged mice was observed, but also thymocyte unresponsiveness to Con A was found in NOD mice [57]. The role of IL-2 has been demonstrated in many autoimmune systems including IDDM in NOD mice [58], [59]. The defect in IL-2 production in NOD mice was mapped to the Idd-3 region [60], [61]. Mice with IDDM susceptibility alleles at *Idd3* have reduced IL-2 levels compared to mice with C57BL/6-derived resistance alleles [62]. Interestingly, in a previous study comparing NOD^B6.Idd3^ mice with NOD mice, a decrease in IL-2 production was not found in NOD mice. The authors determined intracellular IL-2 levels of CD8^+^ T cells after 5 hours stimulation with PMA and Ionomycin [63]. This discrepancy between this report and our study may at least result from the differences in the stimulation itself (PMA and Ionomycin vs. Con A), the stimulation period (5 hours vs. 20 hours), the mouse strain that IL-2 gene derived from (C57BL/6 mice vs. Balb/c mice), and the sample used to examine IL-2 level (cells vs. culture medium). A possible explanation is that IL-2 production in NOD mice might not be able to last for long period (e.g., 20 hours), although we cannot exclude the possibility that the IL-2 level detected in culture medium may be the net result of both production/secretion and utilization/internalization pathways.

Lymphopenia can be promoted by IL-21-induced T cell death through inhibition of IL-7-mediated T cell survival in NOD mice [3], [64]. In addition to NOD mice, it has been shown that a mutation in Gimap5 gene results in lymphopenia and is a prerequisite for spontaneous IDDM in the BioBreeding (BB) rat [65], [66]. In addition, increased lymphocyte apoptosis [67], reduced thymic output [68], and viral infections [69], [70] have been proposed to cause lymphopenia in different autoimmune diseases. Nevertheless, the mechanisms underlying lymphopenia remain elusive. Interestingly, in NOD mice, full disease penetrance only occurs under pathogen-free conditions. In fact, infection with different viruses can delay or even prevent disease in NOD mice [71], [72]. In addition to IDDM, several human autoimmune diseases, such as RA and systemic lupus erythematosus, are occasionally associated with lymphopenia [73]. The fact that lymphopenia occurs occasionally may reflect the “dynamic” process of premature T cell aging as demonstrated in this study. The existence of premature T cell aging in autoimmune diseases has also been demonstrated in several late-onset and early-onset autoimmune diseases, including multiple sclerosis, RA, juvenile RA, juvenile idiopathic arthritis, and myelodysplastic syndrome [12], [14], [15]. Thus, it is tempting to hypothesize that premature T cell aging is a common cause of autoimmune diseases, especially for those in which expansion of autoreactive T cells plays important roles such as primary biliary cirrhosis [74], and that, the faster immune aging occurs, the earlier autoimmunity develops. Nevertheless, further investigation in different autoimmunity models is required to test this possibility.

Although most studies to date use the term “homeostatic proliferation”, rather than LIP, homeostasis of the T cell population is regulated by 2 distinct modes of cell proliferation: slow homeostatic proliferation (HP) driven by low-affinity self-peptide/MHC ligands and the cytokines IL-7 and IL-15 [53]–[56], and rapid spontaneous proliferation (SP) induced by high-affinity interactions with self-peptides or commensal bacterium-derived peptides presented by MHC molecules [75], [76] as well as by the action of the cytokine IL-6 [77]. LIP is a feature of both naïve and antigen-experienced memory T cells [78]–[80], and it has been demonstrated that naïve T cells undergoing LIP can acquire characteristics of memory T cells [81]–[84]. The naïve cells undergoing SP rapidly acquire the memory phenotype, including high CD44 expression and IFN-γ secretion [52], [85]. In addition, SP of naïve cells produces cells that upregulate CD44 and IFN-γ expression to a greater degree than cells undergoing HP, thus generating the majority of damaging autoreactive T cells [52], [85]. It has been shown that if naïve T cells are transferred into syngeneic hosts with irradiation-induced T-cell deficiency, the majority of cells undergo HP, while only a small proportion of cells undergo SP [53], [76]. Proliferating cells with the naïve phenotype include naïve cells undergoing slow HP and likely also include a fraction of cells that have just undergone SP but have not yet acquired the memory phenotype. The LIP of cells with memory phenotype could result from SP of naïve or preexisting memory cells [53], [75], [76] and could also result from HP regulated by IL-7/IL-15 due to reduction in the number of naïve cells.

Our data show that when the percentage of naïve CD4^+^ T cells is equal when comparing NOD and Balb/c mice, the level of LIP in cells with the naïve plus memory phenotype is higher in NOD mice than in Balb/c mice. This difference was due to increased levels of LIP in cells with memory phenotype compared to cells with naïve phenotype (Figure 6B and 6C). In NOD mice, the LIP of memory cells rather than that of naïve cells is significantly and inversely correlated with the extent of lymphopenia. This might be due to the major role that memory T cells play in controlling the LIP of naïve cells [86]. Given IL-7 concentration increases with T cell depletion [87], thus, increasing T cell depletion, as indicated by a lower percentage of naïve cells, leads to more HP, and the presence of the same percentage of naïve cells suggests similarly slow HP. Therefore, our results suggest that, at the same percentage of naïve CD4^+^ T cells, the difference in LIP between NOD and Balb/c mice is due to SP. Since SP is mediated by high-affinity interactions between the TCR and the self-peptide/MHC, our results also in consistent with the presence of autoreactive T cells with high affinity to self-antigens (e.g., self β-cell antigens) in NOD mice. As previously noted, SP can regulate memory phenotype development and production of inflammatory cytokines [77]; therefore, our results suggest SP plays an important role in the pathogenesis of IDDM in NOD mice.

Under lymphopenic condition, where does T cell proliferation occur? T cell proliferation during LIP occurs in T cell area [88]. LIP entry into the T cell areas in the spleen and lymph nodes (LNs), i.e., the periarteriolar lymphocytes sheaths (PALS) in the spleen and the paracortex in LN, is essential for lymphopenia-induced proliferation [88], [89]. The reason may be that, IL-7 and additional factors, including chemokines, are present inside these areas, and the concentration of these factors is presumably increased under lymphopenic conditions [87]. Interestingly, it has been shown that hematopoietic cells are minor contributors to IL-7 [90]. Blocking LIP in peripheral lymphoid organs including spleen inhibited the onset of IDDM in NOD mice. Interestingly, LIP of CD4^+^ T cells in spleen (r = 0.53) correlated better than LIP of CD4^+^ T cells in pancreatic lymph nodes (PLNs) (r = 0.42) with insulitis, although those mice with peri-insulitis had the greatest fraction of CD4^+^ T cells proliferating in the PLNs. Therefore, although may function in different period, both LIP in PLNs and LIP in the spleen play important roles in the development of IDDM in NOD mice. To test our hypothesis, a site where T cell proliferation during LIP can occur (e.g., spleen) should be used. One of the reasons that Balb/c mice were used in this study is that, lymphopenia in total CD4^+^ T cells and naïve CD4^+^ T cells in the spleen has been demonstrated in aged mice [2]. On the other hand, in C57BL/6 mice, also often used as control animals, a significant decrease in the number of naïve CD4^+^ T cells of the spleen is not observed in aged mice [91].

**Figure 7:**
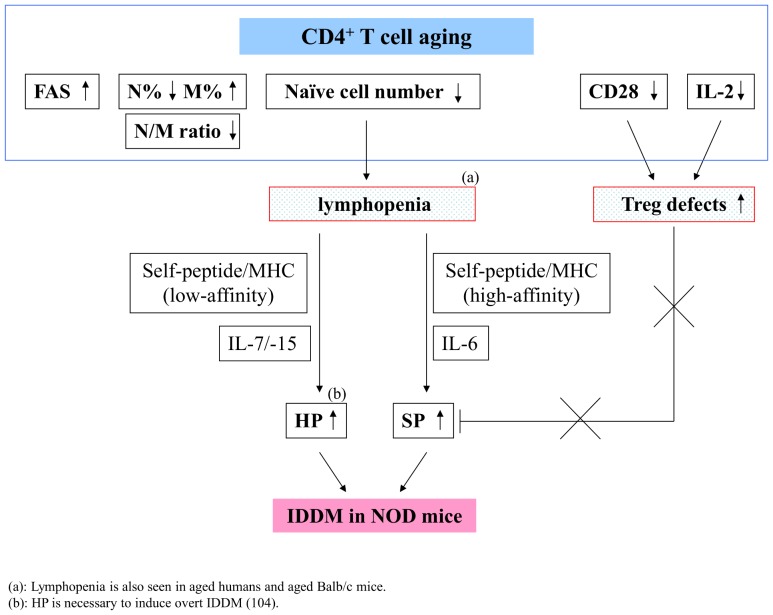
The proposed relationships between premature CD4^+^ T cell aging, lymphopenia, Treg defects and IDDM in NOD mice.

On comparison with control aged Balb/c mice, our results also argue that lymphopenia alone is insufficient for inducing autoimmunity, but rather provides an opportunity for the expansion of autoreactive T cells in autoimmune-prone animals or humans; that is, both lymphopenic status as well as the presence and expansion of autoreactive T cells with high affinity to self-antigens are required to cause autoimmunity in NOD mice. For example, neonates who are characterized as lymphopenic and undergo LIP, do not develop autoimmunity [92]–[95]. First, the expansion of autoreactive T cells is probably due to the Treg defects, given that Tregs can block SP but not HP [85]. It has been shown that IL-2 and CD28 play important roles in the control of peripheral Treg numbers [96]. Because the levels of CD28 and IL-2 are reduced in NOD mice compared to age-matched Balb/c mice (Figure 3D and Figure 4), our results provide further evidence to demonstrate that premature CD4^+^ T cell aging can cause IDDM in NOD mice by causing defects to Treg cells. Thus, these results are consistent with reports demonstrating both lymphopenia and Treg defects are required to induce autoimmunity [9], [10]. Second, in addition to interaction of autoreactive T cells with high-affinity self-peptide/MHC, the cytokine IL-6 may be required to mediate rapid spontaneous proliferation. Tajima et al. reported that IL-6 signaling is crucial for SP under lymphopenic conditions, which subsequently leads to the development of autoimmune colitis [77]. However, whether IL-6 also plays a role in SP to cause a further reduced naïve/memory ratio in T cells, leading accumulation of cells with memory phenotype in NOD mice requires further investigation. The relationships between CD4^+^ T cell aging, lymphopenia and Tregs to cause autoimmunity in NOD mice are proposed in figure 7.

Other inflammatory cytokines that are induced in the diseases process may also play a role in driving the decrease in T cell naïve/memory ratio and maturation of CD4^+^ T cells. It has been shown that IL-6 together with other cytokines, IL-1β, IL-21, IL-23 and TGF-β, are required for the development of Th17 cells in mice [97]. Th17 cells are a subset of CD4^+^ T cells that can secrete IL-17, IL-22, IL-6, and TNF-α, and have been implicated in autoimmune diseases, including multiple sclerosis, lupus, inflammatory bowel disease, RA, IDDM, and experimental autoimmune encephalomyelitis [98]–[101]. Microbiota may also play a role in driving the maturation of T cells since rapid spontaneous proliferation (SP) could be induced by high-affinity interactions with commensal bacterium-derived peptides presented by MHC molecules [76]. The potential differences in gut microbiota may exist in NOD vs. Balb/c mice to mediate IDDM development. It was found that the presence of SFB (segmented filamentous bacteria) as well as a decreased Firmicutes/Bacteroidetes ratio was associated with attenuated IDDM in NOD mice [102], [103].

The difference in LIP between NOD and Balb/c mice was more apparent in age group II, further reflecting the difference between NOD and Balb/c mice with respect to the percentage of naïve cells in this age group. Given that the percentage of naïve CD4^+^ T cells, CD28 expression and IL-2 levels are significantly decreased in age group II NOD mice, it is expected that the LIP in NOD mice of age group II includes both HP and SP (Figure 6D). Indeed, it has been demonstrated that β-islet cell self-reactive CD4^+^ T cells were not sufficient, and IL-7-mediated homeostatic proliferation was necessary to induce overt diabetes mellitus [104]. Conversely, in age group III the total numbers of T cells and naïve CD4^+^ T cells was similar in NOD and Balb/c mice. Since the level of LIP was the highest in age group II of NOD mice, this increase in naïve cells in age group III is likely due, at least in part, to the results of LIP of age group II. Although a reduced number of CD4^+^ T cells was also present in age group IV of NOD mice, the LIP was not significantly higher than that of Balb/c mice. A similar result, obtained using different assay, was also observed in the report of King et al. [3]. One possible explanation for this is that autoreactive cells in age group IV of NOD mice have already reached a state of replicative senescence. Alternatively, it could be due to the fact that Treg defects in age group IV NOD mice are not as severe as those in age group II NOD mice (Figure 4). Interestingly, our results show that the LIP level of NOD mice is significantly higher than that of Balb/c mice only in age group II, when all lymphopenia, reduced IL-2 production and decreased CD28 expression occur, but not in age group I, III or IV, which was characterized by only reduced IL-2 production, decreased CD28 expression or lymphopenia (Figure 3A, Figure 3D and Figure 4). These results might advance what we already knew; that is, the increased LIP of CD4^+^ T cells in NOD mice of age group II might level off with advancing age. However, this high level of LIP may not occur during healthy aging, and it can only be reached during premature CD4^+^ T cell aging in autoimmune-prone condition, in which all lymphopenia, Treg detects, autoreactive T cells, and IL-6 are present, either in early-onset diseases in the young or in late-onset diseases in the middle-aged or aged animals or humans. Taken together, our results suggest that dynamic T cell aging can trigger LIP, thus favoring the contribution of autoreactive T cell expansion to the development of IDDM in NOD mice.

In addition to LIP and Tregs, other T cell defects may also contribute to the pathogenesis of autoimmunity. Interestingly, these defects might also correlate with LIP. For example, the presence of CD4^+^ regulatory T cells plays an important role in the control of the onset of autoimmunity [105]. It has also been shown that the expansion of regulatory CD4^+^ T cells in response to lymphopenia is weaker than that of conventional CD4^+^ T cells in NOD mice [106]. Furthermore, in patients with RA, high levels of TNF-α and IFN-γ are mainly produced by the accumulated CD28^−^ but not CD28^+^ T cells [28], [42]. It is known that CD28 expression on T cells will be gradually lost under replicative stress [46], [107]. Given that reduced CD28 expression has been proposed in RA patients, it was hypothesized that the accumulation of CD28^−^ cells might be due to premature LIP in RA patients [5]. Additionally, lymphopenia has been shown in the RA murine model of K/BxN mice. In this model, spontaneous arthritis could be prevented by inhibiting LIP of autoreactive CD4^+^ T cells [4].

Our results provide important information for the consideration of new therapeutic strategies for patients with IDDM. For example, it has been shown that in the case of islet transplantation in patients with autoimmune diabetes, T cell loss due to the standard immunosuppressive treatment regimen induces expansion of autoreactive memory T cells and thus could contribute to recurrent autoimmunity [108]. Therefore, the outcome of islet transplantation or other therapies can be evaluated and even predicted by monitoring the markers of T cell aging and the expansion of cells with memory phenotypes.

Our results further led us to hypothesize that the factors that could enhance premature T cell aging will promote the onset of IDDM in NOD mice. In contrast, manifestations that could attenuate premature T cell aging (e.g., IL-2) will likely delay the progress of IDDM. It has been shown that immunotherapies that increase IL-2 production/action could correct an immunodeficiency in IL-2 production and prevent IDDM [109]. A possible explanation is that increasing IL-2 can delay T cell aging, restore immune tolerance to β-cells (e.g., through mediation of Treg function), and thus prevent the onset of IDDM.
